# Assay performance verification and reference interval establishment for PIVKA-II on the Roche cobas e 801

**DOI:** 10.1186/s12876-026-05088-7

**Published:** 2026-07-09

**Authors:** Youli Zhao, Zejing Liu, Chunhong Xiao

**Affiliations:** Laboratory Medical Center, The Second Hospital & Clinical Medical School, Lanzhou University, No. 82, Cuiyingmen Street, Chengguan District, Lanzhou, Gansu, 730030 China

**Keywords:** PIVKA-II, Performance verification, Method comparison, Reference interval

## Abstract

**Objective:**

To validate the analytical performance of the Roche cobas e 801 chemiluminescent immunoassay for Protein induced by vitamin K absence or antagonist-II (PIVKA-II), establish age-/gender-specific reference intervals for healthy adults in the Gansu population, and evaluate its concordance with the Abbott Alinity i system currently used in our laboratory.

**Methods:**

Precision (intra-run/inter-run coefficients of variation [CVs]), linearity (0.50-12000 ng/mL), and clinical reportable range (via 10-fold dilution with 80%-120% recovery criteria) were verified following Clinical and Laboratory Standards Institute (CLSI) and Chinese health industry standards. Agreement between the cobas e 801 and Alinity i systems was assessed using Passing-Bablok regression and Bland-Altman analysis (*n* = 173). Reference intervals were established based on 2253 healthy participants (≥ 18 years, permanent residents of Lanzhou). The 2.5th-97.5th percentiles were calculated using a nonparametric method, and decision tree analysis was used to determine age stratification cutoffs.

**Results:**

Intra-run coefficients of variation ranged from 1.36% to 1.57%, and inter-run CVs from 1.13% to 1.16%, all below CLIA’88 total allowable error and manufacturer claims (except for intra-run high-value quality control (QC), which was slightly higher than the manufacturer’s specification). The linear regression equation was Y = 0.99X + 6.34 (R^2^= 0.99), and deviations at all concentration points were < 1/2 Total allowable error (Tea) (11%), meeting linearity requirements. Recoveries after 10-fold dilution ranged from 89.62% to 92.16%, validating the clinical reportable range of 15.30-91533.33 ng/mL. Verification using 20 healthy individuals showed that 100% of results were below the manufacturer’s reference interval upper limit (< 28.4 ng/mL). Method comparison revealed that the intercept 95% confidence interval (CI) (-2.16 to 3.42) included 0, indicating no significant constant (systematic) error, while the slope 95% CI (0.4693 to 0.6925) did not include 1, indicating significant proportional bias. Bland-Altman analysis showed that 96.50% (167/173) of data points fell within the limits of agreement, indicating good agreement between the two methods. The reference interval for males (≥ 18 years) was 9.63–29.10 ng/mL, with no age stratification required. For females, age-stratified reference intervals were established: 9.50–26.30 ng/mL for ages 18–54 years, and 9.42-30.00 ng/mL for ages ≥ 55 years. We recommend a unified reference interval of 9.52–29.10 ng/mL for routine clinical use. Age- and sex-stratified intervals are statistically significant but have modest clinical impact (∼3 ng/mL difference in upper limits).

**Conclusion:**

The PIVKA-II assay on the Roche cobas e 801 system meets laboratory quality requirements for analytical performance and is suitable for clinical application. Due to significant proportional bias compared with the Abbott Alinity i system, system-specific reference intervals are recommended, and the two systems should not be used interchangeably. For results approaching the upper reference limit, clinical follow-up is advised. The established local reference intervals can provide more targeted decision-making support for hepatocellular carcinoma screening.

**Supplementary Information:**

The online version contains supplementary material available at https://doi.org/10.1186/s12876-026-05088-7.

## Introduction

Primary liver cancer (PLC), predominantly hepatocellular carcinoma (HCC), originates from hepatocytes or intrahepatic bile duct cells [1]. The global incidence of HCC is rising, highlighting the critical need for early detection and treatment to improve patient outcomes [2]. Protein induced by vitamin K absence or antagonist-II (PIVKA-II) is a serum biomarker that increases during malignant transformation of hepatocytes. Its concentration correlates with HCC stage and the risk of post-transplant recurrence [3]. According to the Standard for Diagnosis and Treatment of Primary Liver Cancer (2024 Edition), PIVKA-II serves as a valuable early diagnostic marker for HCC, particularly in patients with negative serum alpha-fetoprotein (AFP) levels [4]. Thus, PIVKA-II plays a pivotal role in the diagnosis, differential diagnosis, and prognosis of HCC [5].

PIVKA-II is commonly measured using chemiluminescence immunoassay (CLIA), a method known for its automation, high sensitivity, rapid turnaround, and lack of radioactive contamination. In accordance with the Health Industry Standards of the People’s Republic of China (WS/T), this study aimed to: (1) validate the analytical performance of the new PIVKA-II assay on the Roche cobas e 801 immunoanalyzer (Roche Diagnostics, Mannheim, Germany) in our laboratory; (2) establish a reference interval for PIVKA-II in the healthy population of Gansu Province; and (3) evaluate its consistency with the Abbott Alinity i system (Abbott Laboratories, IL, USA), currently used for PIVKA-II testing in our laboratory.

We hypothesized that the Roche cobas e 801 platform would demonstrate satisfactory performance meeting industry standards, that the established reference interval would be appropriate for the Gansu population, and that acceptable agreement would be observed against the Abbott Alinity i system. The novelty of this study lies in: (i) validating PIVKA-II on the newly launched Roche cobas e 801 platform; (ii) establishing the first reference interval for the Gansu population; and (iii) providing a direct method comparison between Roche and Abbott systems. Collectively, these efforts aim to ensure the accuracy and reliability of the assay for clinical application.

## Materials and methods

### General information

Specimens for performance validation and consistency evaluation were collected from patients and health check-up individuals at The Second Hospital & Clinical Medical School of Lanzhou University between July 1, 2024, and July 20, 2024.

For reference interval establishment, data were retrospectively collected from 2,253 individuals between August 1, 2024, and November 22, 2024. Among these, 110 were healthy volunteers recruited from the hospital’s health examination center. The remaining 2,143 were individuals who had undergone PIVKA-II testing during routine clinical care (inpatient or outpatient settings) but, after rigorous health screening, were determined to have no conditions known to affect PIVKA-II levels. Because PIVKA-II testing is expensive and not included in routine health check-up packages, this retrospective hospital‑based approach was necessary to achieve a sufficient sample size for age‑ and sex‑stratified reference interval analysis. The detailed exclusion criteria applied to all 2,253 participants are listed below:

- History of liver disease (hepatitis, cirrhosis, HCC), diabetes, cardiovascular disease, autoimmune disorders, renal dysfunction, or any malignancy;
- Current use of anticoagulants (e.g., warfarin);
- Chronic alcohol consumption;
- Abnormal liver function tests: alanine aminotransferase (ALT), aspartate aminotransferase (AST), gamma-glutamyl transferase (GGT) above reference limits;
- Positive hepatitis B surface antigen or anti-hepatitis C virus (anti-HCV) antibodies;
- Abnormal renal function or complete blood count;
- Evidence of liver lesions on abdominal ultrasound (if available);
- Hemolysis, icterus, or lipemia exceeding predefined thresholds.

All samples were non-anticoagulated whole blood serum. Each sample was rigorously screened using the following criteria: (1) hemolysis: hemoglobin concentration < 0.5 g/L and no visible red discoloration; (2) icterus: total bilirubin concentration < 20 µmol/L; (3) lipemia: triglyceride concentration < 2.0 mmol/L and no visible turbidity. Samples exceeding any of these thresholds were excluded.

The study was approved by the Institutional Review Board of The Second Hospital & Clinical Medical School of Lanzhou University (approval No. 2023 A-459).

### Instruments and reagents

This study employed two detection systems: the Roche cobas e 801 immunoanalyzer (hereafter referred to as cobas e 801) and the Abbott Alinity i system (hereafter referred to as Alinity i). Both systems used original reagents, calibrators, quality control (QC) products, and consumables supplied by their respective manufacturers. The Abbott PIVKA-II reagents, QC products, and calibrators were manufactured by Abbott GmbH, Germany (production address: 1359-1 Kagamida, Kigoshi, Gosen-shi, Niigata, 959–1695, Japan). The Roche PIVKA-II reagents, QC products, and calibrators were manufactured by Roche Diagnostics GmbH (Sandhofer Strasse 116, 68305 Mannheim, Germany).

For performance validation on the cobas e 801 system, all reagents were sourced from the same batch and shipment to ensure consistency. Specifically, the lot number for the Abbott PIVKA-II reagent was 6094LP50, and that for the Roche PIVKA-II reagent was 75,456,001. This standardization minimized variability and ensured result reliability.

### Performance verification

#### Precision verification

Precision of the PIVKA-II assay was evaluated in accordance with Clinical and Laboratory Standards Institute (CLSI) EP15‑A2 verification protocol [6]. Two levels of quality control (QC) materials were used: low-value QC (target value: 18.2 ng/mL; range: 14.4–22.0 ng/mL; 1 SD: 1.27; calculated CV: 6.97%) and high-value QC (target value: 300 ng/mL; range: 237–363 ng/mL; 1 SD: 21.0; calculated CV: 7.00%). One batch of QC material was tested per day over five consecutive days, with three replicate measurements for each concentration level. For each batch, the mean and standard deviation (SD) were calculated, and inter‑run imprecision was expressed as (SD/mean) × 100%. The overall SD and overall mean across all batches were calculated, and intra‑run imprecision was expressed as (overall SD / overall mean) × 100%. The acceptance criterion was that the observed imprecision should be lower than the manufacturer’s claimed imprecision.

#### Linear range verification

According to the manufacturer (Roche), the declared linear range for the PIVKA-II assay is 0.5-12000 ng/mL. Linearity validation was conducted following the WS/T408-2024 guidelines [7]. The assay was considered acceptable if the deviation at all concentration points was less than the clinically acceptable allowable deviation, set as 1/2 total allowable error (TEa).

Given the current unavailability of external quality assessment (EQA) for PIVKA‑II, an alternative split‑sample comparison was performed in accordance with WS/T 415‑2024 [8]. Based on biological variation, TEa was calculated as TEa = 1.65 × CVA + 1.25 × CVI = 22% [9]. Accordingly, the allowable deviation for linearity verification was set as 1/2 TEa (i.e., 11%).

For validation, one low-concentration clinical sample L (15.30 ng/mL) near the lower limit and one high-concentration clinical sample H (9153.33 ng/mL) near the upper limit of the linear range were selected. These samples were mixed at the following ratios to generate five distinct concentration levels: L, 0.75 L + 0.25 H, 0.50 L + 0.50 H, 0.25 L + 0.75 H, and H. Each concentration level was assayed in triplicate, and the mean values were calculated. The linear range was considered acceptable if the deviation between measured and expected values at all concentration points was less than 11%.

#### Clinical reportable range

The clinical reportable range was verified in accordance with the China National Accreditation Service for Conformity Assessment (CNAS)-GL037 guidance for the verification of quantitative measurement procedures in clinical chemistry [10]. The upper limit of the reportable range is defined as the product of the upper limit of the measurement interval and the maximum allowable dilution factor. To validate this, three high-concentration specimens near the upper limit of the linear interval were selected, with concentrations of 10836.50 ng/mL, 9539.00 ng/mL, and 8844.00 ng/mL. Each specimen was subjected to a 10‑fold dilution using Roche Diluent Universal, as recommended by the manufacturer. Both the original and diluted samples were assayed in duplicate, and the mean concentrations were calculated.

Recovery rates were calculated using the formula:

$$\begin{aligned}\mathrm{Recovery}\;\left(\%\right)=\;&\left(\text{Measured concentration of diluted sample}\;\right. \\& \left./\;\text{Theoretical concentration}\right)\times100\%\end{aligned}$$

where the theoretical concentration was derived from the undiluted sample value divided by the dilution factor. If the recovery rates fell within the acceptable range of 80%-120%, the upper limit of the clinical reportable range was confirmed as the product of the upper limit of the linear range and the maximum dilution factor. This verification ensures the accuracy and reliability of the assay for high-concentration samples, supporting its suitability for clinical use.

#### Reference interval verification

According to the manufacturer (Roche), the declared reference interval for PIVKA-II is < 28.40 ng/mL. The verification was performed in accordance with the Chinese Health Industry Standard (WS/T402-2024) for defining and determining reference intervals in clinical laboratories [11]. As well as the CLSI EP28-A3c guideline [12], twenty serum samples from healthy adults (10 males and 10 females) were randomly collected for verification. Inclusion criteria were as follows: (1) age ≥ 18 years; (2) no history of liver disease (hepatitis, cirrhosis, or hepatocellular carcinoma); (3) normal liver function tests (ALT, AST, and GGT within reference interval); (4) negative for hepatitis B surface antigen and anti-HCV antibodies; (5) no history of alcohol abuse; (6) no evidence of liver lesions on abdominal ultrasound; and (7) no history of malignancy or other serious systemic diseases.

All 20 samples were tested to determine the proportion of results exceeding the declared reference limit of 28.4 ng/mL. If no more than 2 out of 20 samples (≤ 10%) exceeded this limit, the manufacturer’s reference interval was considered applicable to our laboratory. This verification ensures that the reference interval is appropriate for the local population and meets clinical laboratory standards.

#### Comparison of Cobas e 801 and Alinity i

Our laboratory has historically used the Alinity i system for PIVKA-II testing. In this study, the agreement between the Alinity i and cobas e 801 systems was evaluated using Passing-Bablok regression and Bland-Altman (BA) analysis [13, 14]. Passing-Bablok regression is suitable for continuously distributed data with a linear relationship between the two methods. The intercept of the regression equation represents constant (systematic) error, while the slope represents proportional error. The 95% confidence intervals (CIs) for the intercept and slope were calculated to determine whether they differed significantly from the theoretical values (intercept = 0, slope = 1), thereby assessing agreement. Bland-Altman analysis was also employed to compare quantitative results between the two platforms. The mean values of paired measurements from both systems were plotted on the horizontal axis, and the differences between measurements on the vertical axis. The mean difference (d) and the 95% limits of agreement (LoA), defined as d ± 1.96 × SD, were calculated. A narrower LoA indicates higher consistency. Agreement is considered satisfactory if ≥ 95% of the differences fall within the LoA and the range is clinically acceptable. It is important to note that the two systems report results in different units (ng/mL for Roche cobas e 801 vs. mAu/mL for Abbott Alinity i), and no validated unit conversion factor was available. Therefore, the statistical comparisons (Passing-Bablok and Bland-Altman) were performed to evaluate the presence of constant and proportional bias, not to directly compare absolute values. The primary goal was to determine whether results from the two systems could be used interchangeably or whether system-specific reference intervals are required.

#### Establishment of reference intervals for PIVKA-II

Reference intervals for PIVKA-II in local healthy adults were established using the cobas e 801 system in accordance with the CLSI EP28-A3 guidelines [12]. The sample size was determined based on CLSI C28-A3 recommendations, which require a minimum of 120 subjects per subgroup for nonparametric reference interval studies. Accordingly, a total of 2253 participants were enrolled to ensure adequate statistical power for age- and gender-stratified analyses. The criteria for defining healthy adults were identical to the inclusion criteria described in the “Reference Interval Verification” section (Sect. 2.3.4). Reference intervals were derived by analyzing the distribution of PIVKA-II levels in the target population, with the 2.5th to 97.5th percentiles calculated using a nonparametric method. It is important to clarify that the reference intervals established in this study reflect population-based distribution characteristics and differ from estimates of biological variation (including intra- and inter-individual variation) [15]. The latter typically requires a rigorous prospective design with repeated measurements over multiple time points, whereas the present study adopted a cross-sectional approach for reference interval establishment.

#### Statistical treatment

Statistical analyses were performed using Excel 2010 and SPSS 26.0. Count data were expressed as frequencies or percentages. For linearity evaluation, polynomial regression analysis was used to assess the relationship between measured and expected values. For reference interval establishment, statistical analyses were conducted using R software, with primary packages including *readxl*,* rpart*,* ggplot2*, and *dplyr*. Normality was assessed using the Shapiro–Wilk test. Reference intervals (2.5th–97.5th percentiles) were calculated based on data distribution characteristics using a nonparametric method. Decision tree analysis (via the *rpart* package) was employed to identify optimal age cutoff points. Analysis of variance (ANOVA) was used to evaluate differences between age groups, with statistical significance set atα = 0.05. For groups with significant differences, Tukey’s HSD post hoc test was subsequently performed to identify specific pairwise differences.

## Results

### Precision verification

The precision results for the PIVKA-II assay are summarized in Table 1. The intra-run coefficients of variation (CVs) were 1.36% for the high-value QC and 1.57% for the low-value QC. The inter-run CVs were 1.13% for the high-value QC and 1.16% for the low-value QC. Compared with the manufacturer’s claimed values (intra-run: 1.10% for high QC and 1.80% for low QC; inter-run: 5.40% for high QC and 5.90% for low QC), our results were superior, with the sole exception of the intra-run high-value CV, which was marginally higher than the manufacturer’s specification. Notably, substantial improvements were observed in inter-run precision. All CV values obtained in this study were well below the CLIA’88 thresholds (intra-run < 5.00%; inter-run < 6.67%). These findings demonstrate that the PIVKA-II assay exhibits excellent precision and has successfully passed validation.

**Table 1:** PIVKA-II precision verification and manufacturer’s declaration results

QC assays	Average concentration (Mean ± SD)(ng/mL)	Intra-run CV (%)	Inter-run CV (%)	Intra-run CV (%) (Manufacturer)	Inter-run CV (%) (Manufacturer)	CLIA ‘88 Threshold(%)
High value QC	300.27 ± 4.65	1.36	1.13	1.10	5.40	5.00
Low value QC	17.77 ± 0.35	1.57	1.16	1.80	5.90	6.67

*Abbreviations*: *QC* Quality Control, *SD* Standard Deviation, *CV* Coefficient of Variation

### Linear range verification

According to the manufacturer’s instructions, the declared linear range of the PIVKA-II kit is 0.50-12000 ng/mL. The linear regression equation derived from the analysis was Y = 0.99X + 6.34, with a slope of 0.99 and a coefficient of determination (R²) of 0.99. The deviation between measured and expected values at all validated concentration points was less than 11%. These results indicate that the assay meets the required linearity criteria, and the validation was successfully passed, as illustrated in Table 2.

**Table 2:** Linear interval validation data for PIVKA-II (ng/mL)

Dilute	Expected value	Measured concentration				bias
		Average value	1st	2nd	3rd	
L	15.30	15.30	15.40	15.20	15.30	0.00%
0.75 L + 0.25 H	2299.80	2365.00	2380.00	2342.00	2373.00	2.83%
0.50 L + 0.50 H	4584.30	4484.70	4518.00	4456.00	4480.00	-2.17%
0.25 L + 0.75 H	6868.80	6636.70	6641.00	6644.00	6625.00	-3.38%
H	9153.30	9153.30	8802.00	8791.00	9867.00	0.00%

*Abbreviations*: *L* Low value, *H* High value; *1st* First, *2nd* Second, *3rd* Third

### Clinical reportable range

Three samples with concentrations near the upper limit of the linear range were selected: 10836.50 ng/mL, 9539.00 ng/mL, and 8844.00 ng/mL. After a 10‑fold dilution, the theoretical concentration for each diluted sample was calculated as the measured value of the undiluted sample divided by the dilution factor. The recovery rates for the three diluted samples were 89.62%, 90.98%, and 92.16%, all within the acceptable range of 80%-120%. Based on these results, the verified clinical reportable range in our study was 15.30-91533.33 ng/mL. These findings confirm that the assay meets the required performance criteria for dilution integrity, and the validation was successfully passed. Detailed data, including pre‑dilution and post‑dilution concentrations, are provided in Table 3.

**Table 3:** PIVKA-II reportable range results (ng/mL)

Sample	Detected Concentration (post-dilution)	Average value	Theoretical concentration (pre-dilution)	Recovery rate (%)
Sample 1	9590.00	9712.00	10836.50	89.62
	9834.00			
Sample 2	8726.00	8678.50	9539.00	90.98
	8631.00			
Sample 3	8196.00	8150.50	8844.00	92.16
	8105.00			

### Reference interval verification

Serum samples from 20 healthy adults (10 males and 10 females) showed PIVKA-II levels ranging from 12.20 to 22.30 ng/mL. All measured values were below the manufacturer‑declared reference interval upper limit of < 28.40 ng/mL, meaning that 100% of the results fell within the acceptable range. These findings confirm that the manufacturer’s reference interval is applicable to our laboratory, and the validation was successfully passed.

### Comparison of Cobas e 801 and Alinity i

**Fig. 1:**
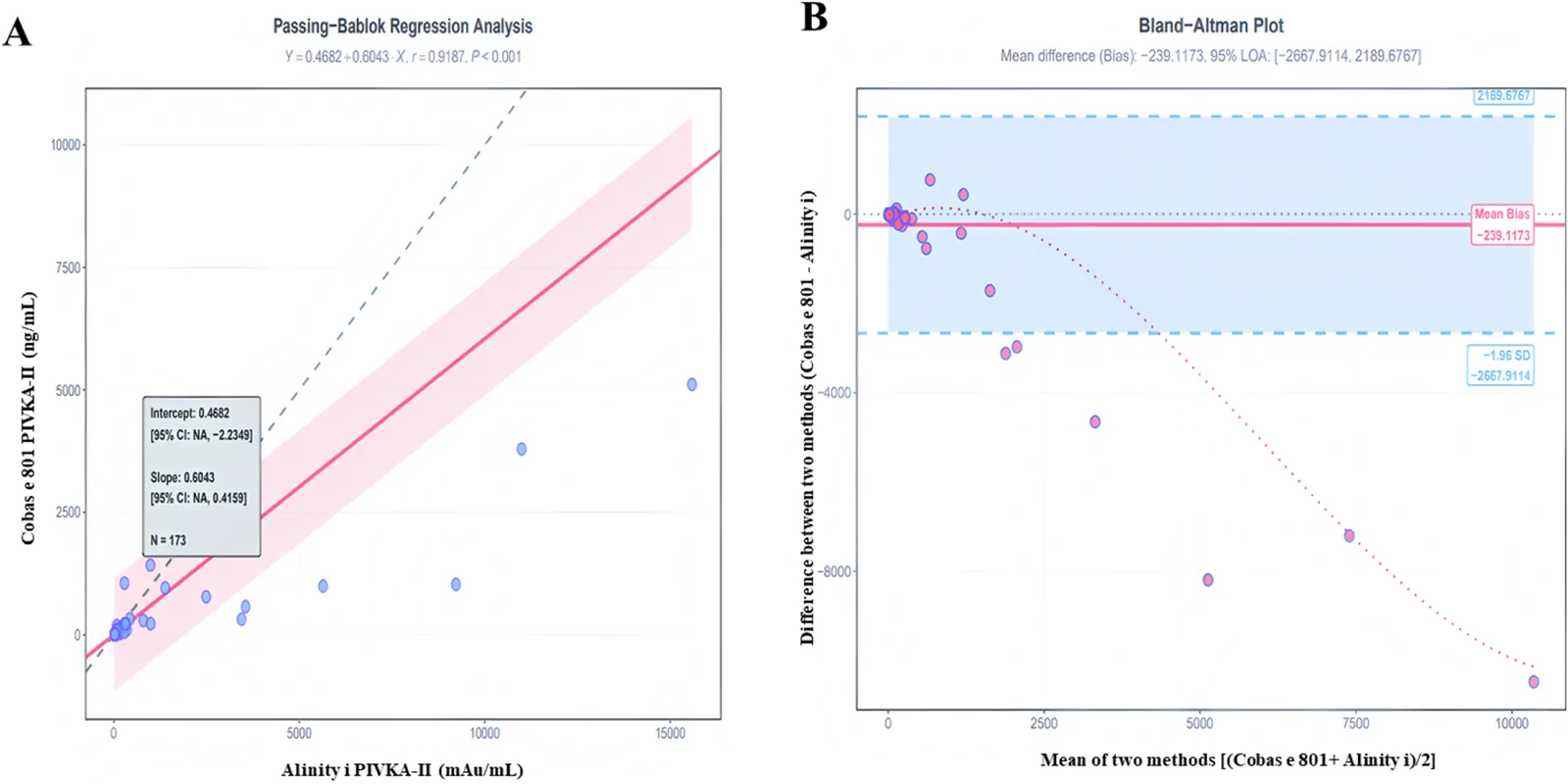
Comparison analysis between Alinity i and cobas e 801 systems. **A** Passing‑Bablok regression; **B** Bland-Altman plot

A total of 173 serum samples were tested for PIVKA-II using both the Abbott Alinity i and Roche cobas e 801 systems. Correlation analysis was performed to evaluate consistency between the two instruments. The linearity assessment (H-test) showed no significant nonlinearity (h stat = 0.858 < h crit = 1.358, *p* = 0.454 > 0.05), indicating a linear relationship between the two methods and supporting the use of Passing‑Bablok regression for agreement evaluation. The 95% confidence interval (CI) for the intercept (-2.16 to 3.42) included 0, suggesting no significant constant (systematic) error. However, the 95% CI for the slope (0.47 to 0.69) did not include 1, indicating significant proportional bias (Fig. 1A). Bland-Altman analysis revealed a mean difference (d) of -239.12 between the two methods, with a standard deviation of differences (SD) of 1239.18. As shown in Fig. 1B, Although 96.50% (167/173) of data points fell within the LoA, the limits themselves were very wide (-2667.91 to 2189.68), indicating poor individual agreement. This suggests that while the two methods rank samples similarly, the absolute differences between paired results are often too large for clinical interchangeability.

### Establishment of reference intervals for PIVKA-II

A total of 2253 participants were enrolled in this study. Table 4 presents the baseline characteristics and distribution of PIVKA-II values across the study cohort. The comparable age distributions between genders supported valid gender-based comparisons. The mean PIVKA-II value in males (16.66 ng/mL) was slightly higher than that in females (15.96 ng/mL), and the difference was statistically significant (*P* < 0.001). However, this difference was clinically modest, as evidenced by substantial overlap in the reference intervals between genders. Age distributions showed moderate dispersion (standard deviation ≈ 12 years in both groups), supporting the feasibility of age-stratified analyses.

**Table 4:** Distribution of gender, age, and PIVKA-II values (ng/mL) in the study population

Gender	Number	Age(year) Mean ± SD	Age range (year)	PIVKA-II values Mean ± SD	Full PIVKA-II range
Female	931	53 ± 12	18–92	15.96 ± 4.59	3.50–39.60
Male	1322	50 ± 12	18–94	16.66 ± 4.75^***^	5.00-39.40

*Abbreviations*: *SD* Standard Deviation

^***^P < 0.001

**Fig. 2:**
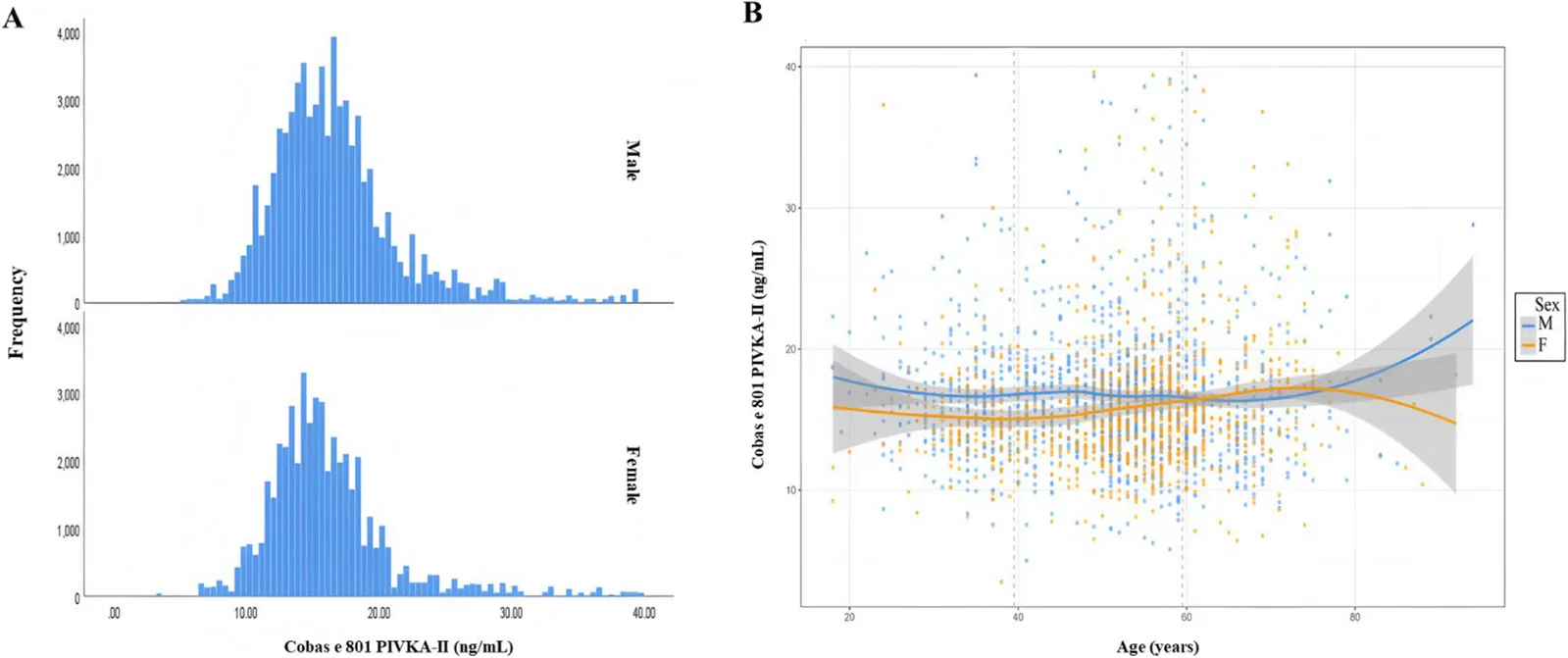
Distribution of PIVKA-II. **A** Histogram showing skewed distribution. **B** Scatter plot with age-related LOESS trends

The Shapiro-Wilk normality test indicated a non‑normal distribution of PIVKA‑II values in both genders (males: *p* = 5.12 × 10⁻²⁷; females: *p* = 1.05 × 10⁻²⁶; both *p* < 0.001). As shown in Fig. 2A, the distribution patterns were skewed for both genders. Figure 2B reveals significant patterns in PIVKA‑II values with respect to age, providing visual evidence supporting the necessity of age stratification. Each point represents one study participant, with blue points for males and orange points for females; point density reflects sample distribution across age groups. Critically, the smoothed curve (LOESS regression) illustrates the age‑related trend: females showed a pronounced increasing trend with advancing age, whereas males exhibited a relatively stable pattern, remaining largely constant across age. The grey shaded area represents the 95% confidence interval (CI), indicating the reliability of the trend estimate.

**Fig. 3:**
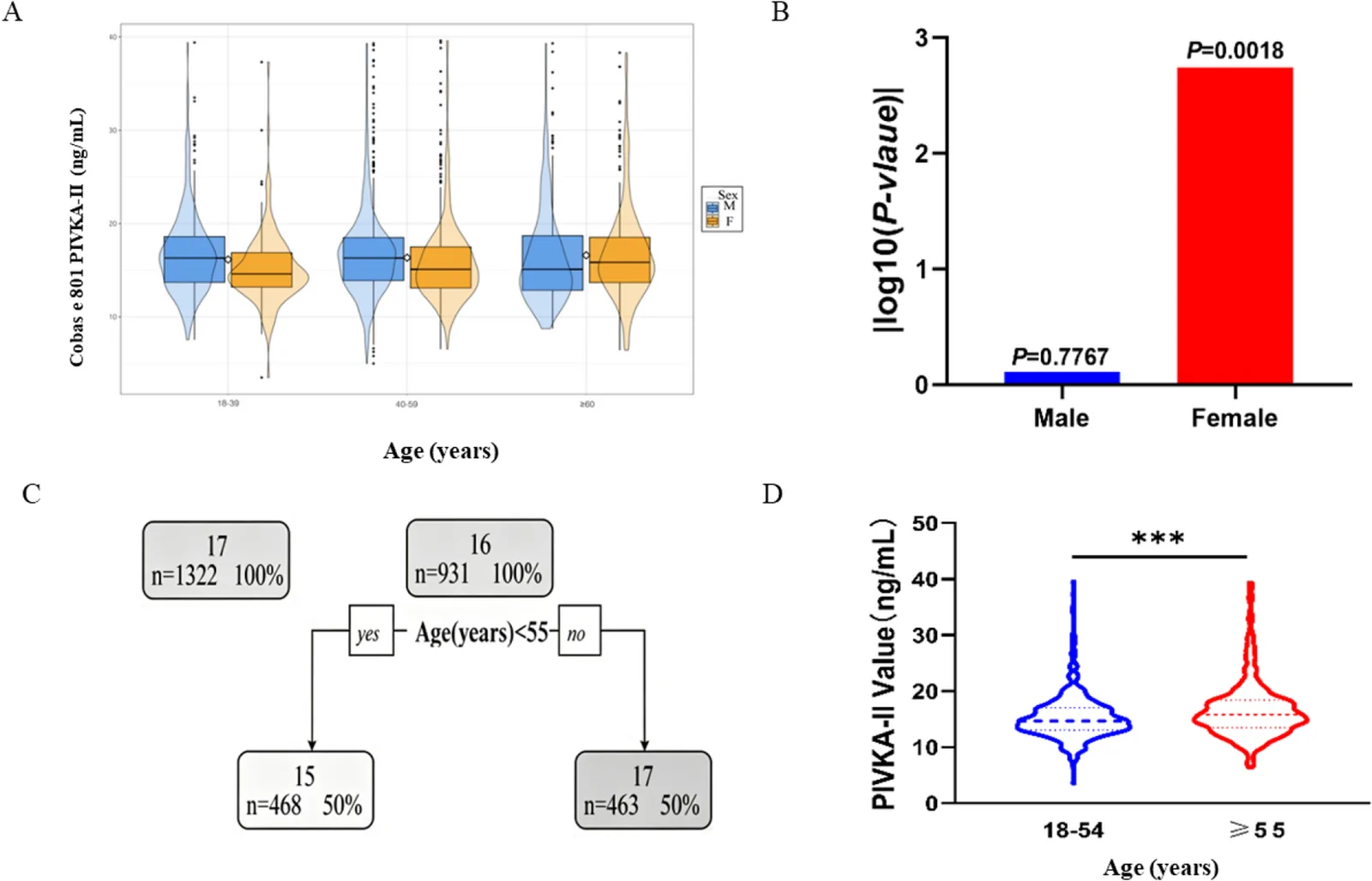
Analysis of PIVKA-II reference interval variations by gender and age group. **A** Combined box and violin plots of PIVKA-II level distribution; (**B**) ANOVA of inter-group age differences; (**C**) Decision tree analysis: male and female PIVKA-II values; (**D**) Tukey HSD post hoc analysis (female subgroups)

Considering that integer age bands are more commonly used in clinical practice, we initially adopted the age groupings of 18–39, 40–59, and ≥ 60 years to investigate the distribution characteristics and intergroup differences of measured values across different age groups. The combined box and violin plot (Fig. 3A) showed no significant differences in PIVKA‑II values among the three male age groups (*p* = 0.777). In contrast, the three female age groups exhibited a distinct pattern of differences, demonstrating a stepwise increasing distribution with advancing age (*p* = 0.002). Meanwhile, the bar chart (Fig. 3B) visually presented the statistical results of the ANOVA test. The y‑axis represents the absolute p‑value after -log₁₀ transformation, where higher values indicate greater statistical significance. The bar height for males was 0.11, which fell below the threshold of 1.30 (corresponding to *p* = 0.05), indicating no significant differences among male age groups and supporting the use of a unified reference interval strategy. In contrast, the bar height for females was 2.74, significantly exceeding the threshold of 1.30, demonstrating significant intergroup differences and necessitating age stratification. Subsequent decision tree analysis revealed that the root node displayed “17 100%”, where “17” represents the mean value at the root node and “100%” indicates that all 1322 male samples converged at the root without age‑related branching (Fig. 3C), further confirming that age has no significant effect on male PIVKA‑II values and that a unified reference interval can be adopted without age stratification. In comparison, the female decision tree exhibited a clear branching structure, with the root node splitting at age 55 years. Figure. 3D shows a significant difference in PIVKA‑II values between females aged 18–54 years and those aged ≥ 55 years (*p* < 0.001).

The 90% confidence intervals for the reference limits are provided in Table 5. Based on the integrated findings from decision tree analysis and statistical testing, a unified reference interval of 9.63–29.10 ng/mL was established for males (≥ 18 years), with no age stratification required. For females, two age-stratified reference intervals were established: 18–54 years and ≥ 55 years. As presented in Table 5, the female cohort comprised 468 participants aged 18–54 years and 463 participants aged ≥ 55 years. Mean PIVKA-II values demonstrated an age-dependent increase. Notably, the ≥ 55 years group exhibited a wider reference interval (9.42-30.00 ng/mL), reflecting greater inter-individual variability within this demographic. The overall reference interval was 9.52–29.10 ng/mL(irrespective of gender and age). Given the small magnitude of differences between subgroups, the unified interval is recommended for routine clinical practice.

**Table 5:** Reference intervals for PIVKA-II values (ng/mL) by age groups for local female subjects

Subgroup	Number	PIVKA-II values (mean ± SD)	Lower limit (90% CI for 2.5th)	Upper limit (90% CI for 97.5th)
Male (≥ 18 years)	1322	16.64 ± 4.83	9.63 (9.62–10.30)	29.10 (26.4–29.2)
Female (18–54 years)	468	15.51 ± 4.10	9.50 (8.41–9.82)	26.30 (24.5–28.5)
Female (≥ 55 years)	463	16.62 ± 5.05	9.42 (8.49–10.40)	30.00 (25.8–32.9)
All (≥ 18 years)	2253	16.41 ± 4.77	9.52 (9.42–9.62)	29.10 (28.00-29.40)

## Discussion

This study confirms that the Roche cobas e 801 PIVKA-II assay meets clinical and industry standards for precision, linearity, and clinical reportable range. A significant proportional bias was observed compared with the Abbott Alinity i system, and unified and stratified reference intervals were established for the Gansu population. Overall, the cobas e 801 system demonstrates stable and reliable performance, supporting its use in early HCC screening and auxiliary diagnosis.

Our precision and linearity results were comparable or superior to those of published PIVKA-II verification studies. For example, Ko et al. (2018) reported intra-run CVs of 3.6%-5.5% for another chemiluminescence platform [16], whereas our intra-run CVs ranged from 1.36% to 1.57%. Our inter-run precision (1.13%-1.16%) was markedly better than the manufacturer’s claimed values (5.40%-5.90%), likely due to stringent reagent standardization and operational protocols. Similar to validations of other tumor markers on the Roche e 801 system, we obtained excellent linearity (R² > 0.99) [17]. To our knowledge, this is the first independent performance validation of the Roche cobas e 801 PIVKA-II assay in a Chinese population, confirming its clinical suitability.

A key finding is the significant proportional bias between the Roche cobas e 801 and Abbott Alinity i systems, despite good correlation and absence of constant error. Importantly, the two systems report results in different units (ng/mL vs. mAu/mL) without a validated conversion factor. Therefore, the Passing‑Bablok and Bland‑Altman analyses presented here are intended only to detect the presence of constant and proportional bias - not to compare absolute values or to imply that results can be mathematically transformed. Despite this limitation, the analyses clearly demonstrate that the two systems are not interchangeable. Such bias is not uncommon across different immunoassay platforms [18] but has important implications for clinical practice and result harmonization. Several technical factors may explain this bias. First, differences in metrological traceability: Roche and Abbott likely use different reference calibration standards or traceability chains for PIVKA-II assignment, leading to systematic differences [19]. Second, differences in reagent formulation: the two platforms differ in antibody specificity (epitope recognition), capture/detection antibody pairs, buffer composition, and signal amplification systems. Even minor differences in antibody affinity or cross-reactivity can produce proportional bias [20]. Third, calibrator matrix effects: the matrix of calibrators (e.g., human vs. animal serum, presence of stabilizers) may affect antibody binding kinetics and recovery profiles [21]. Fourth, subtle differences in detection principles: although both systems are based on chemiluminescence, variations in reaction time, temperature, and signal detection algorithms may further influence quantitative results. Consequently, results from the Roche cobas e 801 and Abbott Alinity i systems should never be directly compared or used interchangeably. Even if a conversion equation were derived, the nonlinear proportional bias and lack of unit harmonization preclude reliable cross‑platform interpretation. Clinical laboratories must adopt system‑specific reference intervals and ensure that patients undergoing serial PIVKA‑II monitoring remain on the same analytical platform throughout follow‑up.

Given the significant proportional bias, we strongly recommend using system-specific reference intervals. The cobas e 801 reference intervals established in this study (males: 9.63–29.10 ng/mL; females: 9.50–26.30 ng/mL for ages 18–54 years, and 9.42-30.00 ng/mL for ages ≥ 55 years) should not be directly applied to Abbott Alinity i results. Laboratories switching systems must re-establish their own reference intervals or derive conversion equations, though the latter may be nonlinear and require careful validation. Furthermore, for patients requiring serial PIVKA-II monitoring (e.g., post-operative HCC recurrence surveillance), the same analytical system must be used consistently to avoid bias that could be misinterpreted as disease progression or treatment response. If a system switch is unavoidable, a parallel comparison study should be conducted to characterize the bias and adjust clinical decision thresholds accordingly [22].

Previous studies reported 95% reference intervals for PIVKA-II in the Chinese Han population of 40.38 mAU/mL (ARCHITECT) [23] and 28 mAU/mL (LUMIPULSE) [24], the latter being consistent with our results. These differences highlight the impact of detection platforms, reagents, and study population characteristics [25]. Consistent with previous studies [26, 27], we observed significantly higher PIVKA-II levels in males than in females, and an age-dependent increase in females. The latter may be attributed to declining estrogen levels during perimenopause and menopause, as estrogen has been shown to modulate hepatic metabolism and suppress hepatocarcinogenesis. However, the clinical magnitude of these differences is modest, with approximately 3 ng/mL difference in the upper reference limit between subgroups. Given that PIVKA-II is indicated as an “aid in diagnosis” of HCC rather than a confirmatory test, such small differences are unlikely to influence clinical decisions regarding further imaging or biopsy referral. Therefore, for routine clinical practice, we recommend using the unified reference interval of 9.52–29.10 ng/mL (irrespective of gender or age). The age- and sex-stratified intervals presented in Table 5 are statistically significant but have limited clinical utility; they should be considered exploratory and may serve as a reference for future mechanistic studies or meta-analyses.

Based on our findings, we propose the following precautions for clinical application: (1) Use system-specific reference intervals: laboratories should not interchange reference intervals across platforms. For the Roche cobas e 801 system, either the unified interval (9.52–29.10 ng/mL). (2) Follow-up for values near the upper reference limit: for patients with PIVKA-II values slightly exceeding the upper reference limit, clinical correlation and follow-up testing are recommended to monitor potential upward trends, rather than making diagnostic decisions based on a single measurement. A rising trend over serial measurements is more concerning than an isolated borderline elevation. (3) Avoid interchanging systems for longitudinal monitoring: as noted above, consistent use of the same system is essential for patients undergoing serial PIVKA-II measurements.

This study has several limitations. First, the reference interval cohort was derived from a single center in Lanzhou, Gansu Province. Lanzhou is an urban center with a predominantly Han Chinese population. Dietary habits (including vitamin K intake from leafy greens), ethnic composition (e.g., lack of representation of Tibetan, Hui, or other minority groups), and environmental factors (altitude, air pollution) in this region may differ substantially from other parts of China. Therefore, the established reference intervals may not be directly applicable to populations with different dietary or ethnic backgrounds. Multi-center validation is needed. Furthermore, the hospital‑derived cohort was not composed of prospectively recruited healthy volunteers. These individuals sought medical care for minor or non‑hepatic reasons and were found free of relevant diseases at the time of testing, which may introduce selection bias. Nevertheless, given the high cost of PIVKA‑II testing, a prospective healthy cohort of this size was not feasible. We therefore consider this rigorously screened hospital‑derived cohort a pragmatic alternative for reference interval estimation, while cautioning against generalization to populations with different healthcare access patterns. Second, we did not assess biological variation (intra- and inter-individual), as this requires a prospective design with repeated measurements over time, which was beyond the scope of this cross-sectional study. Third, the method comparison was limited to 173 samples; although this sample size was sufficient to detect proportional bias, a larger multi-center study would strengthen the evidence. Fourth, while we established reference intervals in healthy individuals, we did not evaluate the diagnostic performance (sensitivity, specificity, ROC curves) of these limits in a cohort of patients with confirmed hepatocellular carcinoma. This is an essential next step before the reference intervals can be recommended for clinical decision-making. Future studies should enroll HCC patients, including those with early-stage and AFP-negative disease, to validate the clinical utility of the unified and stratified intervals. Finally, the absence of an available unit conversion factor between Roche and Abbott systems prevented us from deriving a direct mathematical transformation or assessing whether the proportional bias is linear across the entire analytical range.

Future research should focus on: (1) multi-center validation of the reference intervals established herein, including assessment of clinical sensitivity/specificity in HCC patients; (2) investigation of the biological mechanisms underlying age- and sex-related differences in PIVKA-II levels, particularly the role of estrogen; (3) efforts toward international standardization of PIVKA-II assays, including the development of common calibrators and reference materials; and (4) prospective evaluation of serial PIVKA-II measurements for HCC surveillance, especially in patients with normal AFP levels.

## Conclusion

The Roche cobas e 801 PIVKA-II assay meets laboratory quality requirements and is suitable for clinical use. Due to significant proportional bias versus Abbott Alinity i, system-specific reference intervals are recommended, and the two systems should not be used interchangeably. Results near the upper reference limit warrant clinical follow-up. The established local reference intervals support more targeted HCC screening decisions. Although age- and sex-related differences were statistically significant, their clinical impact is modest. We therefore recommend the unified reference interval (9.52–29.10 ng/mL) for routine clinical use. The age- and sex-stratified intervals are provided as exploratory findings and should not be used for clinical decision-making without further validation.

## Supplementary Information

Supplementary Material 1.

## Data Availability

All data generated or analyzed in this study are included in this article and are available.

## Abbreviations

PIVKA-II: Protein induced by vitamin K absence or antagonist-II (PIVKA-II)
PLC: Primary liver cancer
HCC: Hepatocellular carcinoma
AFP: Alpha-fetoprotein
CLIA: Chemiluminescence immunoassay
CLSI: Clinical and laboratory standards institute
CNAS: China National Accreditation Service for Conformity Assessment
QC: Quality control
TEa: Total allowable error
EQA: External quality assessment
CI: Confidence interval
CV: Coefficients of variation
SD: Standard deviation
LoA: Limits of agreement
ANOVA: Analysis of variance
ALT: Alanine aminotransferase
AST: Aspartate aminotransferase
GGT: Gamma-glutamyl transferase
anti-HCV: Anti-hepatitis C virus
