# Supplementary material for: Assay performance verification and reference interval establishment for PIVKA-II on the Roche cobas e 801
Source: BMC Gastroenterol. 2026 Jul 9;26:602. doi: 10.1186/s12876-026-05088-7 (PMC13632220; doi:10.1186/s12876-026-05088-7)
Supplement: Supplementary file 1 [file 12876_2026_5088_MOESM1_ESM.docx]

**Table S1** PIVKA-II Reference Interval validation results

| Sample number | Gender | Age | PIVKA-II (ng/mL) | Whether or not sth. conforms to |
| --- | --- | --- | --- | --- |
| 1 | Male | 25 | 15.8 | in the interval range |
| 2 | Male | 48 | 22.1 | in the interval range |
| 3 | Male | 39 | 15.5 | in the interval range |
| 4 | Male | 27 | 16.0 | in the interval range |
| 5 | Male | 31 | 18.7 | in the interval range |
| 6 | Male | 55 | 17.6 | in the interval range |
| 7 | Male | 64 | 17.3 | in the interval range |
| 8 | Male | 30 | 19.3 | in the interval range |
| 9 | Male | 49 | 16.2 | in the interval range |
| 10 | Male | 45 | 23.0 | in the interval range |
| 11 | Female | 45 | 15.4 | in the interval range |
| 12 | Female | 34 | 14.6 | in the interval range |
| 13 | Female | 22 | 17.0 | in the interval range |
| 14 | Female | 27 | 13.9 | in the interval range |
| 15 | Female | 28 | 12.2 | in the interval range |
| 16 | Female | 50 | 17.9 | in the interval range |
| 17 | Female | 59 | 15.3 | in the interval range |
| 18 | Female | 66 | 19.8 | in the interval range |
| 19 | Female | 43 | 13.5 | in the interval range |
| 20 | Female | 40 | 22.3 | in the interval range |
